# Recent Cases of Abdominal Surgery*Read before the North Texas Medical Association, June 18, 1902.

**Published:** 1902-08

**Authors:** Henry K. Leake

**Affiliations:** Dallas, Texas


### TEXAS MEDICAL JOURNAL.

ESTABLISHED JULY, 1885.

PUBLISHED MONTHLY.—SUBSCRIPTION $1.00 A YEAR.

VOL. XVIII. AUSTIN, AUGUST, 1902. No. 2.

### Original Contributions.

For Texas Medical Journal.

### Recent Cases of Abdominal Surgery.*

BY HENRY K. LEAKE, M. D., DALLAS, TEXAS.

Acknowledging a request of the chairman that on this occasion I would read a paper on some practical subject, I have selected for discussion the operative treatment of obese women, and, incidentally, the employment of buried sutures, particularly in closing the wound after section of the abdominal wall. Following these remarks I shall append the history of a case, unique in my experience, and certainly a curiosity both from a pathological and operative point of view.

It seems to be axiomatic with surgeons that operations upon obese subjects are comparatively very dangerous, and, therefore, if possible, to be avoided. More particularly is this true in abdominal section cases, such as ventral and umbilical hernias, the removal of densely adherent cysts, old pus tubes, hysterectomies, or, indeed, any condition where, at least with the average surgeon, the operation is difficult and prolonged. I say with the average surgeon, who ordinarily does not possess the dexterity and rapidity in operating that characterizes such men as Jacobs, Doyen, Joseph Price and others, and as was notably the case with the late Lawson Tait, who delighted to impress the spectator with his marvelous genius in "quick deliberation," accuracy and rapid manipulation in just these obese subjects that were approached with such hesitation and timidity in operating, by the average abdominal surgeon.

It is the current belief that obese subjects bear operations very badly. Generally the shock is intense, both from the handling of the intestines and the effects of the anesthetic. It is well under-

*Read before the North Texas Medical Association, June 18, 1902.

stood that obese persons are frequently the subjects of fatty heart, and this, in many cases, if diagnosticated, will prove a serious objection to the performance of any prolonged operation. This latter observation applies with greater force when it is acknowledged that the diagnosis of a fatty heart is extremely difficult, if not impossible; and if we are to assume that it is only the heart that is the subject of the interstitial or of the fibrillary deposit of fat, which is peculiarly dangerous for operation, the problem becomes even more complex, for I apprehend that the diagnosis of either of these conditions ante-mortem is one of the impossibilities even with the most astute and experienced observers in the diagnosis of cardiac diseases.

Moreover, it is certain that the third form of fatty heart, that in which the organ is surrounded and impeded in its action by masses of fat, also is a dangerous condition for the performance of any prolonged operation, albeit the condition could be exactly diagnosticated, a performance not void of doubt and misgiving if an operation is to be based upon its accuracy.

In addition to this, it is known that obese subjects are very liable to nephritis. It matters little with the practical surgeon which form of nephritis is present in the patient proposed for operation, since it is probable that all of these forms are attended by inadequacy of the kidney, or that this may follow immediately upon the operation, although, of course, granular kidney being proved to exist, this would present an insuperable bar to the performance of any operation in which either chloroform or ether must be given. It is probable that inadequacy of the kidney is sometimes a congenital or even acquired peculiarity without structural alteration of the organ, so far as this can be determined by the most scientific examination, thus supporting the assertion of Andrew Clark in his well-known contention. Suppression of urine from the disturbing causes of previous disease, sepsis, surroundings, and other causes, suspected or unknown, may occur previouly to the operation in those whose kidneys are believed, at any rate, to be healthy, or this suppression, complete or incomplete, may follow upon the operation. Such, apparently, was the case in a patient of mine upon whom I recently operated at St. Paul's Sanitarium for suppurative peritonitis consequent upon appendicitis. It was learned that for twenty-four hours previously to the operation there was gradual failure of the kidneys. Just before the operation only a few drams were voided; for the first thirty-six hours after the operation five drams of urine were taken from the bladder, and during the nine days that he lived it was estimated that not more than five or six ounces was the total amount excreted by the kidneys. This patient was a young man of twenty-six, whose habits were asserted to be good, and who never had been suspected of having any urinary disorder whatever. In his case, all other symptoms being ameliorated, his death was ascribed principally to general toxemia resulting from the renal inadequacy that was so pronounced and that could not be overcome by the most persistent and well-directed treatment.

Now, if such a condition intervenes in a patient of average weight and free from previous disease, it would be interesting to know whether or not the obese subject, with apparently healthy kidneys, is more liable, from the predisposing nature of this very obesity, to renal inadequacy, that, more or less closely, may follow upon operative procedures under an anesthetic. From my own experience I am impressed with the belief that this is so, and with especial emphasis in the obese subject having the anemic form of the condition, but from reading and observation I am unable to give any statistics bearing upon this point.

According to Lauder Brunton, chronic nephritis, by the languor that it produces in many cases, is responsible for the accumulation of fat in the system. Chronic nephritis is responsible also in these cases for the blood changes that may be apparent in the anemic form of obesity, and if my impressions are correct, renal inadequacy, without organic renal change, likewise may be responsible for the same systemic conditions. It is a debatable point whether or not renal inadequacy, with normal kidney tissues, may exist, but if there may be congenital absence of one kidney, why may there not be exceptionally congenital disproportion between the amount of kidney tissue and the weight of the body? If this be admitted it follows that renal inadequacy, pure and simple in some cases, may be the cause of obesity and hemic alterations that shall warn us against operations in the class of cases above mentioned. This renal inadequacy, as I have insisted upon elsewhere, is only apparent when tested by some trustworthy method that shall inform us as to the amount of solid matter that is eliminated through the kidneys every twenty-four hours. It is important, then, especially in these subjects, to test the eliminating capacity of the kidneys in this manner, before subjecting any patient to an operation involving large incisions and necessary delay in completing all the surgical details of the procedure.

There are two well recognized forms of obesity—that is, the anemic and the plethoric form; but between these two extremes there are intermediate grades of the condition that will be diagnosticated only after careful microscopic examination of the blood. I may remark that Dr. Wyeth, of New York, who has insisted so cogently upon blood examination in all cases before operating, might have included specifically this observation in his list of hemic conditions that make for or against the performance of any operation. The plethoric form of obesity, as quickly will be inferred, is the most favorable one for operation. In this form the heart and kidneys are comparatively sound, and respiration is less interfered with. Hematosis perhaps is normal, hence the technique of the operation and the anesthetic are borne with comparative safety; and just as the intermediate phases of obesity approach the plethoric, or the anemic form, so will the operation vary in the safety of its performance.

I am aware that Rufus Hall, of Cincinnati, and others, have shown that patients with even nephritis may be so prepared that after a certain time an operation may be performed safely. It is in the so-called emergency cases that my remarks will apply with especial force. But it must be admitted that all forms of obesity are unfavorable for operation, whether with or without complications, as compared with subjects in which the condition does not exist.

Lauder Brunton asserts that: "It is very difficult to give an exact definition of obesity, but in general terms it might be said that obesity commenced when the patient's fat was obviously very much greater than normal, or when it became inconvenient to himself."

"The average height of an Englishman is about five feet eight inches, his weight eleven stone," which, according to a recent act of Parliament, is 154 pounds. "This remains pretty constant till the age of 40 years, when the weight generally began slightly to increase. According to Van Noorden this weight might increase as much as 15 per cent. and still be within the normal limit. When it reached 30 per cent. the person was regarded as stout; from 30 per cent. to 35 per cent. as too stout; from 35 per cent. to 50 per cent. as moderately obese, and above 50 per cent. as very obese."

Now, I have the impression that these figures will apply as well to the average American person, for all the practical purposes of the physician and surgeon, so that we may have a fairly accurate standard by which to judge the safety or extreme danger of operative procedures in patients of the class to which I have referred.

The employment of buried sutures must be regarded as still a moot question, and it is doubtful if for years to come it will be authorized as the invariable custom, even in the closing of wounds judged to be ideally suitable. So much depends upon rigid asepsis, the character of wounds, the material used, the discriminating judgment and skill in the number and placing of sutures, and, what is not always taken into account by operators, the systemic condition of the patient, that results must vary with probable preponderance on the side of failure. That the factors, any one of which may not be omitted, that combine to make of the buried sutures an ideal method of closing wounds, have been realized and practically utilized by surgeons enabled to meet all the requirements, is true, but for the average surgeon throughout the country the practice had best be avoided.

As shown notably by Joseph Price, the through and through sutures, even in the thickest abdominal wall, retain our confidence if they are applied after the approved method that exactly will coapt the parts, leaving no dead spaces, nor undue constriction of the tissues. Of course, catgut, made sterile, if possible, still holds the field against all other materials employed for the purpose, but the future, doubtless, will improve upon its claims and thus establish a greater confidence in the buried suture than at present obtains. While I have endeavored to perfect myself in the use of buried sutures, I have steadily improved, also, in the selection, preparation and skillful adjustment of the through and through suture with increasing satisfaction, both as to the immediate healing of the wound and its future behavior. In 1891, I first began the use of buried sutures in the case of a badly ruptured perineum, with an ugly fistula through the remaining recto-vaginal septum. The latter was divided, and the closing of the perineal rent was done with the Tait incision coapted by four or five rows of buried sutures of sterilized silk-worm gut. The small loops and knots worked out, some into the rectum through sinuses that it was curious to observe finally closed, leaving a fairly good perineum. It may be said that the tissues in close proximity to the rectum are not suitable as a fair test of the retention of the buried suture, but Marcy and Kelly have employed freely the kangaroo tendon in this region, they assert with ideal results. For some years past I have employed catgut frequently as a buried suture in the several layers of the abdominal wall and elsewhere, but latterly, particularly in the closing of the wound after the operation for hernia, my results with buried kangaroo tendon have demonstrated the superiority of this over any other form of suture material. The difference was well shown in a recent case at St. Paul's Sanitarium, where I operated for the radical cure of a large inguinal hernia. The patient, a young man of twenty-five, had increased rapidly in weight for a year past. The incision was of more than ordinary length and depth, the fatty subcuticular layer being fully two inches thick. The sutures, five in number, closing the canal were of the best prepared kangaroo tendon in smaller strands than ordinary; the ends were cut very close to the knots, the fatty layer was sewn accurately with a running line of catgut, while the skin incision was closed by a chain stitch of aseptic silk after the method of Bassini. The pelvis and thighs of the patient were fixed by several layers of plaster of Paris applied over the usual antiseptic dressings. Only a single superficial stitch of silk-worm gut had been placed at the lower angle of the incision near the scrotum. A very small stitch abscess occurred here, the remainder of the long suture line healed beautifully, the silk thread being removed about the ninth day; no suppuration whatever occurring in its track. The patient was troubled with catarrh of the stomach from which he had suffered previously to operation. A bad catheter cystitis also supervened. About the fifteenth day deep-seated suppuration was discovered under the middle of the healed skin incision, and one ounce of pus was removed from a well-defined cavity, involving an area only in the fatty layer in the line of the previously imbedded catgut suture. The deep sutures of kangaroo tendon were never seen, the tissues had healed over them perfectly. The abscess cavity walls were curetted and closed with silk-worm gut sutures; prompt and permanent healing resulted.

It remains to be seen whether or not, as is claimed by some, the kangaroo tendon after one month will be removed by absorption, or at an indefinite future period be extruded by a suppurative process; but in a case in which it was employed similarly two years ago I have had no report of suppuration, although I recently have learned of the patient's condition being normal in every particular.

However, it is proved to my satisfaction, as well as to that of others, that fatty tissue is the least favorable to the absorption, or permanent retention, of any form of buried suture material, no matter how well this is placed. The lobular character of such tissue prevents the uniform obliteration of all dead spaces on its surface, and its vitality comparatively is low, but this latter varies with the individual; an observation that found expression in the oft-quoted phrase of an old nurse of Weir Mitchell: "Some fats are fast, some fats are fleeting, but cod liver oil fat is soon wasted"; consequently, as naturally would be expected, the destinies of buried sutures will vary with their environments, both as to the cellular organization of these and their peculiar vital endowments. At any rate, the buried suture in fatty tissue has a very problematic future, and, therefore, if possible, should be avoided in the closing of all wounds where fat is much in evidence throughout the field of operation.

Illustrating the points I have raised in the preceding remarks, I shall give, now, the brief histories of several cases that recently have occurred in my practice:

CASE 1. For this operation I am indebted to Dr. A. C. Graham, of Dallas, who often renders me valuable counsel and assistance:

Mrs. R., age 57; 5 feet 2 inches in height; weight 150 pounds; married 35 years. Had two children, the last one born 30 years ago, since which time she has had several miscarriages. When Lawson Tait was on a visit to Boston, in 1884, she consulted him with respect to her condition. Mr. Tait diagnosticated a fibroid tumer of the uterus and advised immediate operation that was not performed. Before I examined her she had had uterine hemorrhage, more or less, for twenty years, and had been under the care of many physicians, including a homeopathic physician, for the past five years.

She was classified as moderately obese, with a large pendulous abdomen, owing to enormous accumulation of fat within its walls and in the omentum. She was sallow faced, very nervous, and low spirited. She was somewhat breathless on exertion. Examination of the urine was negative. There were no murmurs in the heart, but the first as well as the second sound was very indistinct. The radial pulse was small and compressible.

The uterus was enlarged to the size of about the third month of pregnancy, with several fibroids that were discovered readily. She was bleeding from the uterus.

Four days later it was considered possible to do a vaginal hysterectomy, and this was accomplished with some difficulty, the uterus being removed without its section. Recovery was uneventful, the patient, in fairly good condition, leaving my private hospital at the end of the fourth week. Twelve days afterward Dr. Graham was called to see her and discovered a very movable coccyx, that probably resulted from the great distension owing to the withdrawing of the tumor mass, although the patient averred that it was done several weeks previously to the operation, by a physician who gave her agonizing pain on dilating the vagina with a large speculum.

The coccyx was removed by operation, after which, with the exception of some nervousness and indigestion, the patient has remained well.

CASE 2. Mrs. W., Plano, Texas; age 44; apparent height 5 feet 5 inches; weight 200 pounds; the mother of 13 children, the youngest being about five years of age. Her complexion was not ruddy; her breathing was embarrassed on much exertion, and the arterial tension was low. Examination of heart and urine negative. She is classified as very obese and in the intermediate grade. This patient consulted me with respect of an umbilical hernia the size of a large orange, and that protruded through an abdominal wall, showing great rolls of fat. The hernia had existed for sixteen years. She was subject to spells of colicky pains in the abdomen that gave her and the family physician much anxiety. Following the advice of Kelly and others, I declined to operate, and advised her to wear an abdominal belt. This advice was ignored.

About six months after her visit to me I was summoned hurriedly to Plano, where at midnight I operated on her for acute strangulation. The sac contained an enormous mass of thickened and adherent omentum that enclosed about four inches of the small intestine of a dark purple, almost gangerous, color. After a wait of at least half an hour and the application of warm salt water cloths, the bowel resumed its normal color and was returned to the abdominal cavity. The thickened omentum was tied off in four sections with catgut, the mass cut away in healthy tissue, and the stump dropped. The redundant sac, fibrous ring, excess of fat and skin were retrenched freely, and the atropied recti muscles for about one-third their width bordering the enlarged hernial opening, were dissected out and an attempt made to unite broad muscular surfaces with matress sutures of silver wire according to the plan of Kelly, but this was abandoned on account of the extreme tension required to perfect the method. Silver wire through and through sutures were then applied as directed by Joseph Price, with satisfactory coaptation of all the abdominal layers. The sutures were removed about the ninth day, the wound having healed without suppuration. I understand that the patient left her bed in four weeks, and recently has visited in this city. I have had no information of a return of the hernia. Including the half an hour delay above mentioned, the patient was two hours and twenty minutes under the anesthetic, skillfully administered by Dr. Lynch, of Plano. Drs. Jasper and Mendenhall were admirable assistants.

Case 3. Mrs. H. For this patient my thanks are due to Dr. Pace, of Dallas, under whose supervision she was operated at my private hospital.

Widow for three years past; married 16 years; two children; deceased; age 48; height 5 feet 2 inches; weight 147 pounds; menses ceased one year ago. Classed as too stout, but of the plethoric type. Heart and kidneys normal.

In 1900 she had a lacerated perineum repaired and ventro-fixation done for prolapse of the uterus. Suppuration occurred in the abdominal wound, resulting in much diastasis of the recti muscles, between which a hernial protrusion appeared, covered only by peritoneal, fat, and skin layers. This gradually grew larger until it had now reached the size of an orange, but the bowel and omentum were not adherent to the sac wall. Surrounding the hernial opening the walls of the abdomen were pendulous with large amount of fatty tissue.

The operation was performed as in Case No. 2, with the exception that the recti muscles were brought into apposition by a continuous line of catgut. The silver wire sutures were removed on the tenth day, but on the twelfth day pus emerged through the middle of the skin incision. The pus cavity, which involved perhaps three loops of the catgut in the muscular tissue, rapidly healed. This suppuration seemed not to affect the integrity of the muscle union, although it gave me much anxiety. The hernia apparently is cured perfectly, there being no evidence whatever of its return. The cicatrix is firm, and all the abdominal layers are in their normal relations.

Case 4. Mrs. T. Referred to me by my friend Dr. Allen, of Dallas; age 30; weight 132 pounds; married two years; never pregnant. In 1899 she was operated upon three times in Chicago: first, for pelvic adhesions; second, for the removal of the right ovary; third, for the removal of the left ovary, the last operation taking place in September of that year. Dr. Dodds, of Chicago, was the operator, assisted, on one occasion, by my acquaintance Dr. Franklin Martin, an eminent gynecologist. From just below the umbilicus to the pubis there was a wide area of scar tissue, much resembling that following a deep burn. This unsightly cicatrix was very tender on the slightest pressure. The patient suffered from paroxysms of agonizing pain within the abdomen over an area corresponding to the skin cicatrix, but radiating far outside of this. These pains were much aggravated by standing, walking, or, indeed, exertion of any kind whatever. At the lower end of the abdominal incision there was a hernial protrusion the size of a walnut, and this was increasing constantly in volume. The operation proved to be tedious and difficult on account of omental and bowel adhesions that were cautiously separated with fingers and knife. These adhesions existed for a large area around the previous abdominal incisions. Some of these were present as large bands densely organized, particularly was this the case at one point where a knuckle of the ileum was adhered to the abdominal wall. The parts were cleared finally of all adhesions, and the omentum, which had been subjected to much handling, was returned, with some misgivings, to the abdominal cavity, and carefully laid over the intestines. All redundant tissues, including the scar tissue and the skin, were retrenched, the much retracted muscles exposed and sewn together with four interrupted sutures of kangaroo tendon, between which were inserted sutures of chromacized catgut. Through and through sutures of silk-worm gut, previously introduced through the entire thickness of the abdominal wall, were now tied, and completed an ideal coaptation of the parts. The latter were removed on the ninth day, when union was found to be perfect without suppuration. The patient left the hospital at the end of the fourth week, all pains having vanished, with every prospect of permanent relief.

Case 5. Mrs. P. Through the kindness of Dr. Watkins, of Stone Point, Texas, this patient entered my private hospital March 17, 1902. Age 39; height 5 feet 7 inches; weight 245 pounds; married about 20 years; three children, twins at last birth, four years ago. She is of the markedly plethoric type of obesity; complexion very ruddy; heart impulse strong and regular; no heart murmurs; radial pulse volume and tension good; kidneys normal; some urates passed, but no albumen nor sugar. While the muscular system is well developed, fat is superabundant everywhere, particularly in the abdominal walls.

Situated to the right of the nmbilicus, and in contact with it, was a deeply seated mass, the size of a billiard ball, hardish, and lobulated, with an aparently well defined pedicle. The mass could be grasped and pushed from side to side with the fingers. The diagnosis lay between lipoma and an old umbilical hernia with thickened omental contents. At the operation it proved to be the latter. The parts were dealt with as in Case No. 2, excepting that it was found possible to bring the muscles together with buried kangaroo tendons. On twisting the silver wire and tying a few superficial stitches of silk-worm and catgut, the abdominal layers were observed to be well coapted and presenting an artistic appearance. A single superficial stitch at the lower end of the incision suppurated, otherwise when the silver wires were removed on the eleventh day union was found to be complete. This patient was under an anesthetic for one hour and a half, and was put to bed without any symptoms of shock. Wearing an abdominal belt, she left the hospital, in good condition, at the end of the fourth week.

Case 6. Mrs. C. My friend, Dr. Staples, of Wiley, Texas, brought to me this patient. She was placed in bed at the St. Paul's Sanitarium at about 1 p. m. March 22, 1902, when the following notes were taken by Dr. Freedman, the resident physician:

"Widow for thirteen years; is large and very fat; weight 202 pounds; low stature; mother of 11 children, the youngest 14 years old. Skin and mucous membranes pale; temperature 99; pulse 76, small volume and low tension; heart impulse weak. Until five years ago menstruation was normal as to intervals, quantity and duration; after this menstruation came at intervals of three weeks until two years ago, since when flow has been continuous, often passing clots; pain in lower abdomen, at times severe. Has been able to attend to her household duties most of the time, although confined to her bed at intervals. Six weeks ago severe flooding came on and during the last week she has suffered constant, intense pain and nausea, and has had fever.

"Abdominal wall flacid, but enormously fat; tenderness in hypogastric region. Patient placed under chloroform. Uterus enlarged to about the size of the third month of pregnancy; its surface is uniformly smooth and semi-elastic. There is little, if any, movement on strong pressure. Vagina capacious, cervix high up, but admitting the index finger with the hand introduced half way into the vagina; cervix is ballooned out; at the internal os there is felt a softish mass that is believed to occupy the entire uterine cavity. On withdrawing the examining finger this is found to be covered with a large quantity of muco-pus having a bad odor.

"Diagnosis: A soft, degenerating myomatous tumor."

Portions of the growth were removed and submitted to Dr. Shelmire for microscopical examination. This gentleman concurred in the diagnosis. Next morning, at eleven o'clock, consultation was held with Dr. Graham, to whom was given a brief history of the case. Dr. Freedman submitted the following urinary analysis: "Color, light straw; specific gravity, 1022; reaction, acid; albumen very marked; sugar, none; sediment, slight, creamy; not examined for casts." The propriety of the operation was discussed with Dr. Graham, Dr. Freedman and the surgical nurse being present. It was decided finally that the patient could not be improved by further delay, and the operation was proceeded with. She was taken to the operating room at 11:30 a. m. and returned to bed at 2 p. m., one hour having been consumed in the technique of the operation.

First, the cervical lips were sewn together with strong silk, after which the abdomen was opened by a long incision and panhysterectomy done in the usual manner. The operation was difficult, owing to the great thickness of the abdominal wall, the shortened and thickened broad ligaments, and limited working room within a comparatively small pelvis. The removal of the uterus was completed in a thoroughly satisfactory manner, the abdominal incision being closed as in the hernia cases described above—that is, through and through sutures passing entirely through the abdominal wall, with buried kangaroo tendon for the recti muscles. During the latter part of the operation, and thereafter, the patient was in profound shock, from which she never fully rallied, and died at 7 o'clock the same evening.

Examination of the specimen discovered the fact that the uterine cavity, or what was left of it by the advancing mass of the tumor, was filled with a foul-smelling, muco-purulent material. The tumor structure itself, although showing signs of invasion by the suppurating process, which had begun on the remaining wall of the uterus and the surface of the tumor, had not yet begun to show ulceration, although its lowest portion was softish and certainly would have become necrotic within a very short time. It showed, also, from its size and basal connections that the vital powers of the patient would not have been equal to an elimination of the mass, either by its gradual expulsion by uterine contractions conjointly with, or without, a necessarily slow suppuration, or sloughing of the mass. From this point of view, some operation for the removal of the tumor seemed urgently indicated. Curetting or morcellment, the latter a very uncertain and dangerous procedure in tumors situated as this one was, could not safely have been performed. In two similar cases, over twenty years ago, I attempted the curetting operation after forcibly dilating the cervix, the base of the tumors could not have been thoroughly removed, for suppuration continued, the patients dying finally of septicemia not unlike that observed in puerperal cases. On tumors situated lower down, however, on several occasions, I have divided the cervix with large incisions and peeled the tumor from within its capsule, but these operations were attended with great risk and difficulties and were followed invariably by a mild septicemia that, however, resulted fatally in not a single patient. In the case reported, such an operation, in my opinion, could not have been carried out successfully. My last comment upon this case is that it was selected badly, and that, possibly, operation should have been declined; notwithstanding this, it is certain that life was only abridged by its performance a short period. Moreover, it seems certain that delay would not have sufficed to make the operation any the less unnecessary or dangerous.

I shall close my paper by granting the request of a member of this society that I should report an interesting case that occurred in my practice in 1900, some details of which had come to his knowledge. In all my reading and experience I recall not an exact parallel of this case:

Mrs. K. called at my office on July 22, 1900, and desired a prescription for "flowing too much," a condition that had existed only for the three weeks previously. She made the following statement: "I am forty-six years of age; married for twenty years, and have had four children, aged 18, 15, 11 and 5 years, respectively. Never have miscarried, and up to the beginning of the present hemorrhage always have been perfectly regular. I am certain that I never have missed a period except when pregnant, and never have had an abnormal period of any kind. Excepting during my labors, I never have suffered with pelvic pains, nor have I had any fainting spells whatever; and now I have a tired feeling only." Average height, build and weight; complexion bronze-like, suggesting Addison's disease; demeanor strikingly cheerful; bowels regular; appetite good; not nervous.

On July 24th, at her home she was examined under chloroform with the following results: Dark blood was issuing from the os uteri; the uterus was retroverted and somewhat enlarged; bimanual palpation discovered a hard irregular shaped body above the pubis, but deep in the pelvis. It seemed to be about the top of the bladder and could be pressed down so as to be felt by the fingers in the vagina. It could be displaced well to either side by moderate pressure, but on removing this quickly resumed its original site above the bladder. Free or encysted stone, or some form of growth within the bladder, was suspected. The anesthetic was withheld, her husband sent for and questioned concerning any bladder symptoms that, at any time, had been complained of. He knew very little, if anything, about her physical condition, but demanded that if anything was in the bladder it should be removed immediately. Dr. Montgomery was sent for instruments. On his return, surgical anesthesia was induced, the urethra rapidly dilated, and the index passed into the bladder. The before mentioned body was felt, apparently springing from the anterior wall of the viscus, but the mucous membrane smoothly covered it. Still uncertain, I made a large vesico-vaginal fistula, admitting three fingers, and held its lips apart with retractors, thus affording a good inspection of the cavity of the bladder, and a round projection from its anterior wall. On each side of the projection the mucous membrane was seized with separate forceps and pulled down into full view to the vaginal fistula opening. This gave no clue to its nature. The mucous membrane, with underlying exudate, between the forceps was then incised over the contour of the mass and dissected laterally, thus laying bare the surface of the mass, the true character of which was now suspected. Blunt dissection with knife handle and finger cautiously advanced, enucleated the mass to which lithotomy forceps was applied and forcible extraction accomplished, first into the bladder, through the vesico-vaginal fistula into the vagina and out through the vulvar opening. The specimen showed it to be the lime-incrusted skeletal remains, consisting of the skull, thorax, and both femurs of an ectopic fœtus of perhaps three months' growth, the soft tissues and part of the bony frame work having been absorbed.

The cavity above the bladder was large, a glass drainage tube traversing the bladder was passed into this cavity from the vagina through the vesico-vaginal fistula, the vagina packed with loose gauze beyond and in front of the tube, and the operation was complete.

Free drainage was maintained for three weeks, when the glass tube was removed; the patient left her bed one week afterwards. Seven weeks subsequently, the vesico-vaginal fistula not having healed, it was closed by operation, perfect union resulting. The patient is now and since the operation has been in perfect health.

I will not extend this paper, already too long, by speculating as to the pathology of the conditions that in my opinion would explain all the circumstances of this remarkable case, but will leave the subject for your discussion, if you are so disposed.

